# Exogenous hydrogen sulfide gas does not induce hypothermia in normoxic mice

**DOI:** 10.1038/s41598-018-21729-8

**Published:** 2018-03-01

**Authors:** Sebastiaan D. Hemelrijk, Marcel C. Dirkes, Marit H. N. van Velzen, Rick Bezemer, Thomas M. van Gulik, Michal Heger

**Affiliations:** 1 Department of Experimental Surgery, Academic Medical Center, University of Amsterdam, Amsterdam, The Netherlands; 2 Philips Research, Eindhoven, The Netherlands; 3 Department of Anesthesiology, Laboratory of Experimental Anesthesiology, Erasmus University Medical Center Rotterdam, Rotterdam, The Netherlands; 4 Department of Translational Physiology, Academic Medical Center, University of Amsterdam, Amsterdam, The Netherlands

## Abstract

Hydrogen sulfide (H_2_S, 80 ppm) gas in an atmosphere of 17.5% oxygen reportedly induces suspended animation in mice; a state analogous to hibernation that entails hypothermia and hypometabolism. However, exogenous H_2_S in combination with 17.5% oxygen is able to induce hypoxia, which in itself is a trigger of hypometabolism/hypothermia. Using non-invasive thermographic imaging, we demonstrated that mice exposed to hypoxia (5% oxygen) reduce their body temperature to ambient temperature. In contrast, animals exposed to 80 ppm H_2_S under normoxic conditions did not exhibit a reduction in body temperature compared to normoxic controls. In conclusion, mice induce hypothermia in response to hypoxia but not H_2_S gas, which contradicts the reported findings and putative contentions.

## Introduction

Hibernation is a hypometabolic state characterized by a regulated decrease in core body temperature (*T*_b_) (i.e., hypothermia) towards ambient temperature (*T*_a_) and consequent reduction in oxygen (O_2_) consumption and carbon dioxide (CO_2_) production. It is engaged by several mammalian species^1^ to protect the organism from (environmental) stressors such as extreme cold, hypoxia^2,3^, and starvation^4,5^ and ultimately death.

The regulated decrease in *T*_b_, which is termed anapyrexia, encompasses the downmodulation of the ‘internal thermostat’ outside of the thermoneutral zone^6–10^. The thermoneutral zone constitutes a temperature range in which heat production (from basal metabolism) is in equilibrium with heat loss to the environment. The organism functions best when the *T*_b_ resides in the thermoneutral zone, but engages anapyrexia as a coping mechanism. How the anapyrexic signaling is biochemically and physiologically regulated and how the ‘internal thermostat’ is circumvented is largely elusive and hypothetical, but the ultimate outcome is unequivocally a state of hypometabolism. The natural purpose of the hypometabolism is to temporarily realign energy needs with reduced energy/O_2_ supply under conditions of stress in order to sustain life under circumstances that could otherwise have lethal consequences.

The state of cold hypometabolism is believed to be a result of systematic deviation from homeothermy, which in turn is caused by a reduction in or cessation of metabolism. The resulting hypothermia assists, or propagates, the hypometabolic state in accordance with Arrhenius’ law. This law states that the rate of chemical reactions (i.e., metabolism) decreases when the temperature decreases^11,12^. Consequently, both the consumption of substrate (in this case O_2_) and the formation of product (in this case CO_2_, toxic metabolites such as lactate, and reactive O_2_ species) are reduced during hypothermia, as has been confirmed in natural hibernators during hibernation in terms of expired CO_2_^1^. The alignment of metabolic demand with supply as well as the decreased formation of cytotoxic metabolites confer sustenance of life and cytoprotection in the stress-exposed organism.

In line with the above, mimicking these natural phenomena in non-hibernators such as humans by *artificially* inducing hypometabolism holds tremendous potential in medicine, aviation and space travel, and sports. An artificially induced hypometabolic state has been hypothesized to impart similar protective effects on otherwise stressed cells. Accordingly, numerous studies have focused on identifying agents that are capable of inducing hypometabolism in non-hibernating mammals (i.e., anapyrexic agents), which have yielded 5′-AMP^13,14^, DADLE^15,16^, 2-deoxyglucose^5,17^, thyronamines^18,19^, and exogenous hydrogen sulfide (H_2_S)^20^ as potential anapyrexic agents. Of these, exogenous H_2_S has received the most attention in the last few years in response to the Science publication by Blackstone *et al*.^20^. However, our experiments in mice, which duplicated the experiments by Blackstone *et al*.^20^, revealed that exogenous H_2_S does not induce hypothermia at normoxic conditions. Instead, the hypothermia observed in the experiments emanates from a hypoxia-induced anapyrexic response, which is a natural response in mice to hypoxic stress^2,3^. The results are described in this paper and addressed in the context of artificial hypometabolism.

**Figure 1:**
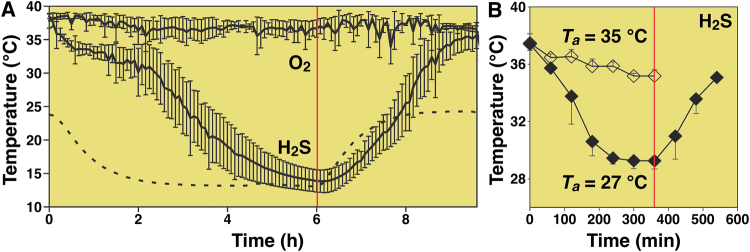
Previously reported temperature effects of H_2_S. Temperature effects of inhaled H_2_S gas (80 ppm) on the *T*_b_ of mice as a function of exposure time as reported by Blackstone *et al*.^20^ (**A**) and Volpato *et al*.^21^ (**B**). In (**A**) mice were exposed to 80 ppm H_2_S and 17.5% O_2_ (*n* = 7) or 17.5% O_2_ (*n* = 4) for 6 h, followed by a recovery phase at 17.5% O_2_ in both groups (6–10 h, right part of red vertical line). The *T*_a_ was decreased during the exposure phase (dotted line). In (**B**) similar experiments were performed as in (**A**) but at fixed *T*_a_s of 27 °C (closed diamonds, *n* = 3) or 35 °C (open diamonds, *n* = 4). The 6-h H_2_S exposure phase was followed by a 3-h recovery phase in air at a *T*_a_ of 27 °C (6–9 h, right part of red vertical line). Data modified from^20,21^.

Exogenous H_2_S has been proposed to induce hypometabolism that is associated with a state of suspended animation^20^. Mice that were subjected to a gas mixture composed of 17.5% O_2_, 80% nitrogen (N_2_), and 80 ppm H_2_S exhibited a 22 °C reduction in *T*_b_ (Fig. 1A) after 4 h of exposure, yielding a *T*_b_ that was slightly above the *T*_a_ of 13 °C. At this point CO_2_ production and O_2_ consumption had decreased by approximately 90%, suggesting that the animals had reached a state of hypometabolism by anapyrexia. Moreover, this state was reversible inasmuch as all metabolic parameters reverted to baseline within 4 h after the exposure to H_2_S was abrogated. During this recovery period the *T*_b_ also gradually restored to baseline at a *T*_a_ of 24 °C. In another study by Volpato *et al*., inhalation of air containing 17.5% O_2_ and 80 ppm H_2_S induced similar anapyrexic effects in mice at a *T*_a_ of 27 °C as well as 35 °C (Fig. 1B) ^21^, altogether suggesting that inhaled H_2_S reduces the *T*_b_ to *T*_a_ levels.

The mechanism behind exogenous H_2_S-induced suspended animation^20,21^ is generally ascribed to the direct inhibition of oxidative phosphorylation^22^ and consequent histotoxic hypoxia. Because of is its high membrane permeability, H_2_S is readily delivered to tissues via the circulation where it transgresses cell membranes and localizes to various intracellular organelles, including mitochondria^23^. H_2_S binds cytochrome *c* oxidase (complex IV) in the electron transport chain in a reversible and noncompetitive fashion. As a result, H_2_S prevents O_2_ binding to cytochrome *c* oxidase and thereby interferes with the reduction of O_2_ to water. Concurrently, H_2_S interferes with the production of adenosine triphosphate (ATP) by ATPase due to H_2_S-induced perturbation of electron transfer and proton gradient over the mitochondrial inner membrane^24,25^. It should be noted, however, that H_2_S-mediated histotoxic hypoxia has never been proven to directly translate to H_2_S-induced hypothermia. Similarly, experimental evidence that H_2_S triggers a downward shift of the thermoneutral zone directly remains at large.

Although the hypometabolic effects of exogenous H_2_S seem convincing, the putative mechanism for the hypometabolic state induced by exogenous H_2_S, i.e., cytochrome *c* oxidase inhibition^22^, may not account for the observed effects. As H_2_S is a toxic, irritant gas^22^, inhalation is known to provoke epithelial damage in the upper^26^ and lower respiratory tract^27,28^ in rats and pulmonary edema in pigs^29,30^. The pulmonotoxicity of exogenous H_2_S may therefore be associated with hypoxemic hypoxia.

Hypoxia, on the other hand, is a very potent inducer of anapyrexia, hypothermia, and hypometabolism and, thereby, of suspended animation^3^. Several hibernating and non-hibernating mammalian species, including mice, exposed to different degrees of hypoxic atmospheres (i.e., *F*_i_O_2_ 5–10%) immediately drop their *T*_b_ to enter a reversible state of hypometabolism^31–34^. The hypothermic effects of hypoxia are known to be caused by downward adjustment of the ‘internal thermostat,’ and involve the preoptic anterior hypothalamus (POAH), as has been demonstrated in thermobehavioral experiments in rodents^2^. Consequently, we proposed that the hypothermia in exogenous H_2_S-exposed mice, which constitutes a hallmark feature of hypometabolism, emanated from the combination of mild hypoxia (17.5% O_2_) and inhalation of H_2_S gas, and not the exogenous H_2_S gas per se.

## Results

**Figure 2:**
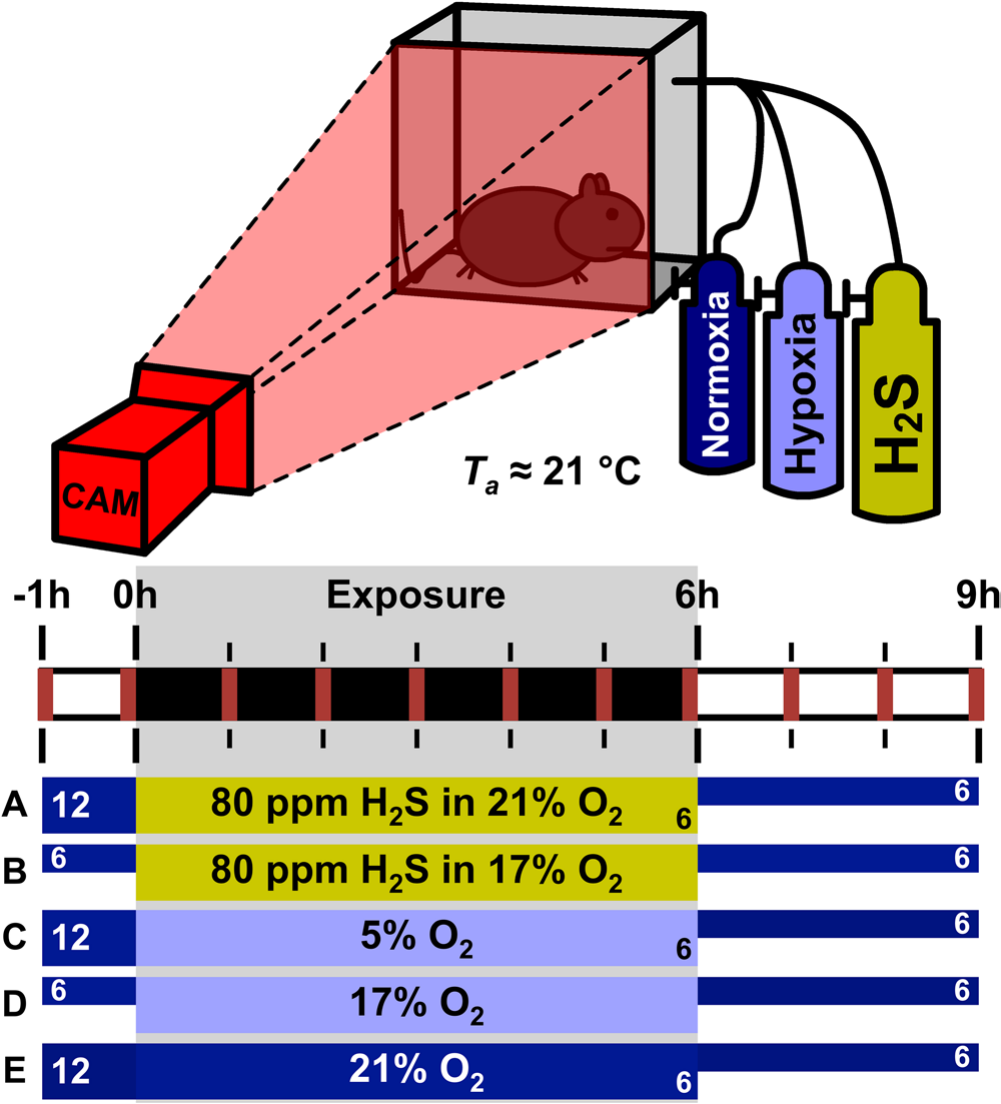
Schematic illustration of the experimental setup and design. The animals were allocated to one of the following experimental groups: (**A**) 80 ppm H_2_S in 21% O_2_ and 79% N_2_ (H_2_S in 21% O_2_ group, N = 12); (**B**) 80 ppm H_2_S in 17% O_2_ and 83% N_2_ (H_2_S in 17% O_2_ group, N = 6); (C) 5% O_2_ and 95% N_2_ (5% O_2_ group, N = 12); (D) 17% O_2_ and 83% N_2_ (17% O_2_ group, N = 6); and (E) 21% O_2_ and 79% N_2_ (normoxia group, N = 12). The experiments were performed at a mean ± SD *T*_a_ of 21.2 ± 0.6 °C, measured with a thermistor. During the whole experiment the mice were solitarily housed in a custom-built airtight cage and recorded with a thermographic camera (CAM) for 10 min every hour (red markers) for skin temperature- and locomotor activity analysis. After 1 h of baseline 21:79% O_2_:N_2_ exposure, each mouse was exposed to a gas mixture (**A–E**) for 6 h that was passed through the airtight cage, after which 6 of the animals in each group were allowed to recover at 21:79% O_2_:N_2_ for 3 h. The other 6 animals of group (**A**,**C** and **E**) were sacrificed for another study.

To test the hypothesis that exogenous H_2_S-induced hypothermia emanates from hypoxia and not H_2_S, we performed experiments in 48 female C57BL/6 mice using a similar approach as was employed by Blackstone *et al*.^20^. The experiments, which are outlined in Fig. 2 and the Materials & Methods section of this paper, encompassed the following groups: (A) 80 ppm H_2_S in 21% O_2_ and 79% N_2_ (H_2_S in 21% O_2_ group; N = 12 mice); (B) 80 ppm H_2_S in 17% O_2_ and 83% N_2_ (H_2_S in 17% O_2_ group; N = 6 mice); (C) 5% O_2_ and 95% N_2_ (5% O_2_ group; N = 12 mice); (D) 17% O_2_ and 83% N_2_ (17% O_2_ group; N = 6 mice); and (E) 21% O_2_ and 79% N_2_ (normoxia group; N = 12 mice). The effects of exogenous H_2_S and a hypoxic atmosphere on *T*_b_ at a *T*_a_ of ~21 °C were measured non-invasively with a thermographic camera and the locomotor activity of the animals was quantitated with dedicated motion analysis software.

**Figure 3:**
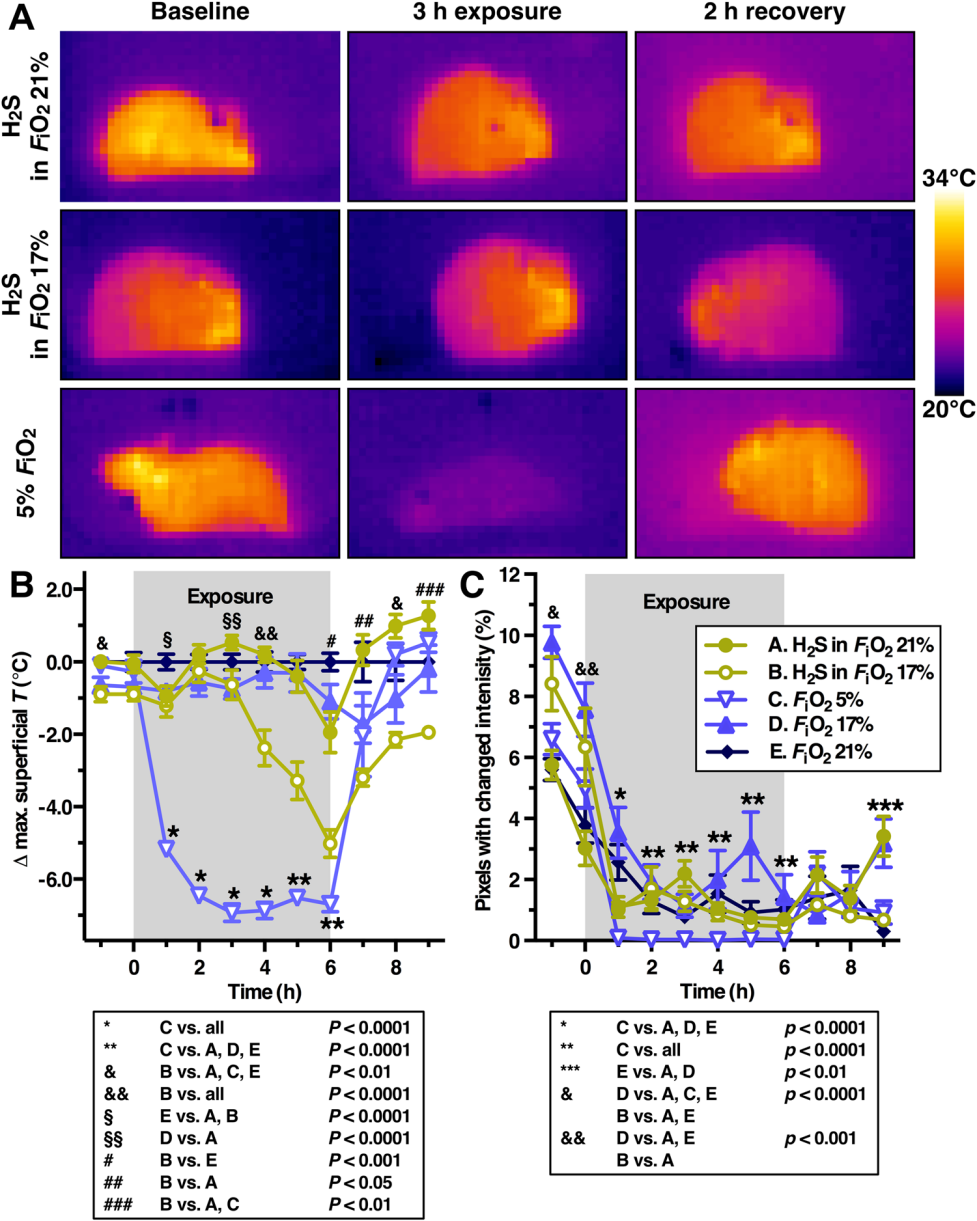
Temperature and locomotor effects of H_2_S compared to hypoxia. (**A**) Thermal images of H_2_S-exposed mice in 21% O_2_ (top row), in 17% O_2_ (middle row), and hypoxia-subjected mice (bottom row) before (baseline), during, and after (recovery) exposure. The color of the animals reflects their temperature (scale bar). (**B**) The difference between the maximum superficial temperature (Δ max. superficial *T*) of the hypoxia (*F*_i_O_2_ 5% and 17%) and H_2_S (*F*_i_O_2_ 17% and 21%) groups versus the normoxia group (*F*_i_O_2_ 21%) was plotted as a function of time before exposure (up to 0 h), during exposure (0–6 h), and after exposure (6–9 h). (**C**) Mouse mean locomotor activity per time point per group plotted as a function of time before exposure (up to 0 h), during exposure (0–6 h), and after exposure (6–9 h). Locomotor activity was derived from temporal changes in pixel grayscale intensity as described in the online supplemental information. In (**B**) and (**C**) the means ± SEM are plotted for N = 12/group (group **A**, **C** and **E**) or N = 6/group (group **B** and **D**) up to 6 h, and for N = 6/group from 6 to 9 h. Statistically significant intergroup differences are displayed under the corresponding plots.

As shown in Fig. 3 and Supplemental Video S1, hypothermia and reduction in locomotor activity only occurred in mice subjected to hypoxic conditions. 5% O_2_-exposed animals immediately dropped their *T*_b_ to approximately 2 °C above the *T*_a_ (Fig. 3B, *P* < 0.0001) and reduced their locomotor activity to nearly nil compared to the H_2_S in *F*_i_O_2_ 21% and normoxia groups (Fig. 3C, *P* < 0.0001) during the entire exposure period. The exogenous H_2_S in 21% *F*_i_O_2_ group did not differ from the normoxia group during 6 h of 80 ppm H_2_S gas exposure in neither superficial temperature nor locomotor activity. At 3 h of exposure, however, animals in the H_2_S in 17% *F*_i_O_2_ group started to drop their *T*_b_ to approximately 4 °C above *T*_a_, in contrast to *F*_i_O_2_ 17%-exposed control animals (Fig. 3B, *P* < 0.0001). Alleviation of the hypoxic conditions during the restoration phase resulted in complete reversal of the superficial temperature to baseline levels within 1 h in the *F*_i_O_2_ 5% group, which is in agreement with previous reports^20,21^ (Fig. 1). During the 3 h of restoration at normoxic atmosphere, the H_2_S in *F*_i_O_2_ 17% -exposed animals remained hypothermic and only restored *T*_b_ to the level of the *F*_i_O_2_ 17% and 21% control groups at 9 h (Fig. 3B, *P* < 0.01). Mice in the H_2_S groups exhibited some discomfort during H_2_S exposure, as evidenced by the cringed posture, which occasionally concurred with vigorous locomotion (Supplemental Video S1).

Peripheral vasodilation is one of the cooling mechanisms that is autonomically regulated in response to a mismatch between the *T*_b_ and the internal thermostat (i.e., *T*_b_ > thermoneutral zone)^3,35,36^. Peripheral vasodilation is integral to anapyrexia^3^, which enables cooling. The cooling process is in turn facilitated by the blockade of thermogenic effectors and the enabling of peripheral vasodilation^36–38^. Therefore, the extent of peripheral vasodilation was determined by measuring the change in tail temperature at baseline and at approximately 4 min after initiation of H_2_S- or hypoxia exposure.

**Figure 4:**
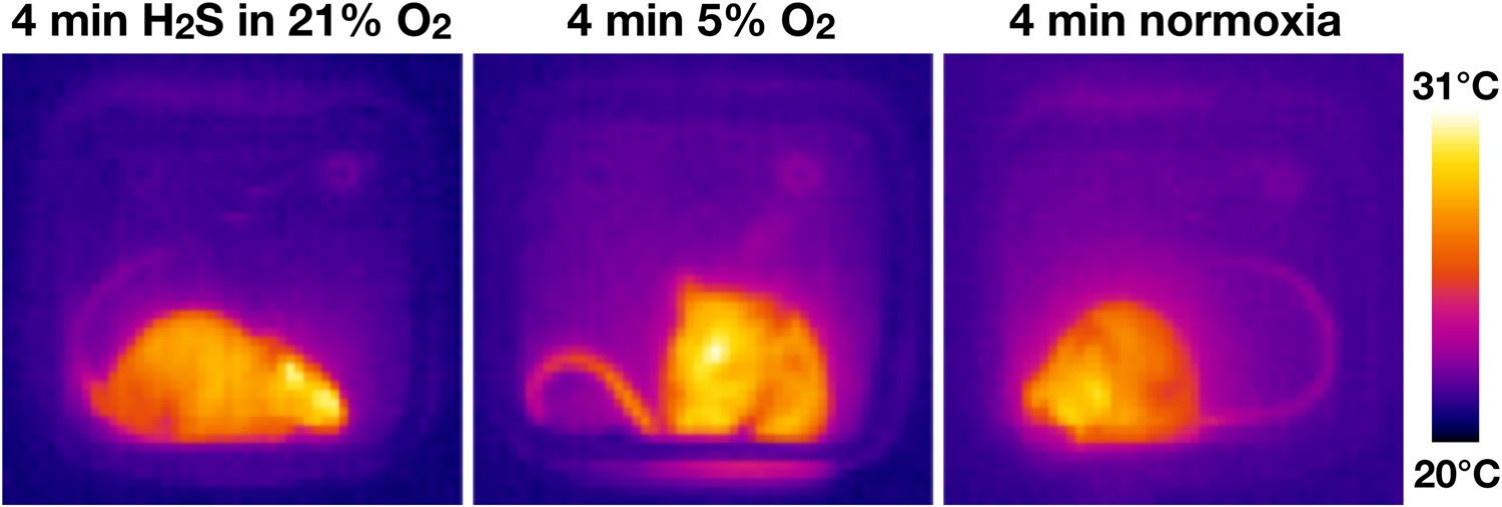
Tail temperatures in H_2_S and hypoxia-exposed mice. Representative thermographic camera images of mice approximately 4 min after initiation of exposure to H_2_S in 21% O_2_ (left), 5% O_2_ (middle), or normoxia (right). Yellow indicates a high surface temperature (31 °C), blue indicates a low surface temperature (20 °C) as indicated by the scale bar. Note the difference in tail temperature (on the basis of yellow intensity) of the hypoxic animal versus the H_2_S gas-exposed and normoxic animals. The warmer tail in the hypoxic mouse is indicative of peripheral vasodilation; a cooling effector that is induced by anapyrexia.

The tail of 5% O_2_-exposed animals warmed up right after the start of exposure (+2.1 ± 0.5 °C, N = 3, *P* < 0.05 versus the H_2_S group, unpaired student’s *t*-test), while the tail of H_2_S in 21% O_2_-exposed animals (−1.3 ± 1.1 °C, N = 3) and normoxia-exposed animals (+0.1 °C, N = 1) did not exhibit changes in temperature (*P* > 0.05, unpaired student’s *t*-test) (Fig. 4). These results provide compelling evidence for the induction of peripheral vasodilation by hypoxia but not exogenous H_2_S, and hence for hypoxia-mediated anapyrexic signaling. The absence of a vasodilatory response in the exogenous H_2_S group is in agreement with the surface temperature data, which encompassed an absence of hypothermia (Fig. 3).

## Discussion

Based on the experimental evidence, namely *T*_b_, tail temperature, and locomotion, it can be concluded that inhalation of H_2_S gas at 80 ppm in a native atmosphere of 21% O_2_ and 79% N_2_ does not induce hypothermia in mice, which contradicts what has been reported previously^20,21^. Hypoxia, on the other hand, is a very potent inducer of hypothermia that, given the peripheral vasodilation observed in the tail vasculature, may comprise part of an anapyrexic response^2,3^. The subclinical thermal effects of mild hypoxia, however, are potentiated by combined 80 ppm H_2_S gas exposure.

One consistent finding in mouse studies on the pharmacological induction of hypothermia is that the animal’s *T*_b_ or surface temperature approximates the *T*_a_ and subsequently enters a plateau phase that is sustained in the vicinity of *T*_a_. Regardless of what actually caused the hypothermic signaling in the experiments by Blackstone *et al*.^20^ and Volpato *et al*.^21^, the *T*_b_ was in all instances downmodulated to a depth at which the *T*_b_ was more or less in equilibrium with the *T*_a_, irrespective of the magnitude of the *T*_a_ (i.e., 13 °C, 27 °C, or 35 °C). The same pattern was observed in our experiments (*T*_a_ = 21 °C), suggesting that the hypothermia may have been mediated via a common mechanism. Moreover, this decline-plateau pattern suggests that the cooling process is passive once the thermogenic effectors have been shut off. The cooling is halted upon reaching a thermodynamic equilibrium where *T*_b_ = *T*_a_, i.e., a point at which the organism is not equipped to cool further. Unlike under normophysiological circumstances, where *T*_b_ is tightly regulated via engagement of cooling effectors or thermogenic effectors^35,39^, the hypothermic state seems to sustain itself through passive heat transfer only.

The main differences between the results of Blackstone *et al*.^20^, Volpato *et al*.^21^, and our results are the rate of cooling and subsequently the time required to reach the plateau phase (*T*_b_ = *T*_a_). The cooling rate was approximately 1.3 °C/h and 4.0 °C/h in the experiments of Volpato *et al*. and Blackstone *et al*., respectively, whereas in our experiments the cooling rate was approximately 5.3 °C/h. The convergence of *T*_*b*_ with *T*_*a*_ required ~6 h in the study of Blackstone *et al*.^20^, ~4 h in the study of Volpato *et al*.^21^, and 2 h in our study (Fig. 3). The same animal species with similar animal weights were employed in all studies. Hence, it is unlikely that these discrepancies arose from differences related to physical laws such as Galilei’s square-cube law^40^, the implication of which is that animals with a large body surface:mass ratio (i.e., small animals) cool faster than animals with a small body surface:mass ratio (i.e., large animals)^1^. The discrepancies in cooling rate also did not emanate from differences in metabolism in accordance with Kleiber’s law, which states that small animals exhibit a relatively higher metabolic rate to maintain euthermia compared to larger animals^1,41^.

In light of the finding that exogenous H_2_S is not an inducer of hypothermia, the question that remains to be answered is “why did Blackstone *et al*. and Volpato *et al*. observe hypothermia in H_2_S-exposed mice?” Volpato *et al*. was able to reproduce the hypothermic effects of 80 ppm H_2_S of Blackstone *et al*. Consequently, we do not question the methodology and validity of their results. In our opinion, the answer lies in the hypoxic conditions that were induced by the combination of subatmospheric *F*_i_O_2_ and the various mild forms of exogenous H_2_S-induced hypoxia. The 3.5% lower *F*_i_O_2_ versus native atmospheric *F*_i_O_2_ (17.5% versus 21%, respectively) is, in itself, not sufficient to trigger anapyrexia in mice, unless such mild hypoxic conditions are exacerbated by exogenous H_2_S. In line with our results obtained in the 17% *F*_i_O_2_ groups, the exacerbation likely occurred in the experiments by Blackstone *et al*. and Volpato *et al*. for four possible reasons. First, as explained in the Introduction section, H_2_S can induce histotoxic hypoxia by inhibiting cytochrome *c* oxidase and corollary ATP production, resulting in reduced metabolic supply (energy). Consequently, the organism is forced to adapt its metabolic demand to survive by means of e.g., hypothermia (Arrhenius’ law). Secondly, H_2_S can limit the binding of O_2_ to hemoglobin’s O_2_ binding sites^42^, thereby causing O_2_ affinity hypoxia^14^. Thirdly, H_2_S reduces cardiac output through its deregulatory and negative chronotropic effects on cardiac rhythm^21,28^, which leads to circulatory hypoxia^43^. Fourthly, H_2_S is pulmonotoxic^26–28^ and may impair pulmonary O_2_/CO_2_ exchange and the extent of O_2_ saturation, which in turn may aggravate the circulatory hypoxia caused by the cardiovascular effects. In addition, based on *ex vivo* experiments, H_2_S seems to play an essential role in hypoxic pulmonary vasoconstriction^44^. Therefore, administration of exogenous H_2_S to the lungs may further compromise pulmonary blood flow during hypoxic conditions, which can augment hypoxemic hypoxia. Accordingly, all these forms of H_2_S-mediated hypoxia may add to the mild hypoxia caused by subatmospheric *F*_i_O_2_ levels and culminate in a hypoxic state that is considerable enough to trigger anapyrexia. As addressed in Dirkes *et al*.^45^, circulatory hypoxia is sensed through carotid bodies located in the carotid artery^46,47^ that, under non-hypometabolism-inducing, hypoxic conditions, relay arterial O_2_ tension (*P*_a_O_2_)-related information to the brain. The brain subsequently (hyper)activates certain physiological functions to remediate the hypoxia^48^, which include panting^49–53^ and tachycardia^53,54^. How this is blocked during the induction of anapyrexia is currently unclear.

Endogenous H_2_S as well as intracerebrally administered exogenous H_2_S analogues inhibit the ventilatory and thermal response to hypoxia in the hypothalamus and brain stem. Contrastingly, microinjection of Na_2_S (H_2_S precursor) in the anteroventral preoptic hypothalamus of rats potentiates hypothermic signaling by hypoxia, but does not alter *T*_b_ under normoxic conditions^55^. Microinjection of the endogenous H_2_S production inhibitor amino-oxyacetate in the sympathetic excitatory rostral ventrolateral medulla of rats attenuates hypoxia-induced hypothermia^56^. As H_2_S passes the blood-brain barrier freely, central effects of inhaled H_2_S could have contributed to hypoxia-induced anapyrexia via the hypothalamus or brain stem,^22^ albeit an unequivocal mechanistic explanation remains warranted in light of the contrasting results.

In the experiments of Blackstone *et al*. and Volpato *et al*., *T*_b_ was determined by telemetry devices that record the core temperature (i.e., intra-abdominal temperature). In our experiments, the superficial temperature was determined. We believe that this approach is valid for the purpose of this study inasmuch as we were interested in temperature trends as a function of exposure time and gas composition, and not the real *T*_b_ per se. Since all groups were thermographically analyzed in the same manner, the resulting data yield credence to our conclusions. Moreover, the use of thermographic imaging has some benefits over intra-abdominal temperature determination, such as the determination of thermoregulatory vasoactivity by tail temperature measurement (Fig. 4).

Although this paper focused on the hypometabolic properties of H_2_S gas, several animal studies on the effects of liquid H_2_S analogues NaHS and Na_2_S have been published. After inhalation, H_2_S gas diffuses freely across the alveolar membrane and enters the blood as predominantly HS^−^ and H_2_S^22^. Accordingly, intravenous administration of solubilized H_2_S precursors/analogues is believed to follow the same pharmacodynamics as administration through inhalation, only without the detrimental effects on local pulmonary physiology and toxicity. The hypothermic effects of NaHS and Na_2_S in small as well as in large animals have been reviewed before^57^. Continuous administration of NaHS is assumed to induce hypothermia in anesthetized rats, although these studies lack essential control groups^58,59^. The evidence considering the hypothermic and hypometabolic effects of NaHS in large animals has been conflicting: in a pig study a small hypothermic effect was observed following 8 continuous hours of NaHS administration^29^, whereas in several other studies in pigs^45,60^ and sheep^61^ such hypothermic effects were not reproducible. The differences between the effects of H_2_S in small and large animals have been contemplated by Dirkes *et al*. and are explained by the inability of large animals to lose heat sufficiently due to the low body surface:mass ratio^45^.

In this paper, the tail temperature was used as a measure of central activation of peripheral cooling mechanisms (i.e., peripheral vasodilation), as has been used before in the determination of thermoregulatory peripheral vasoactivity in pyrexic mice^38^. However, as reviewed by Liu *et al*., H_2_S has biphasic effects on the vascular tone: at low concentrations H_2_S induces vasoconstriction and at higher doses vasodilation is induced, as evidenced in mouse and rat aortic tissue^62–64^. Consequently, the absence of thermoregulatory vasodilation and a consequent increase in the tail temperature of 3 animals (Fig. 4) could also be a direct vasoconstrictive effect of low-dose H_2_S. Nevertheless, H_2_S-induced vasoconstriction is unlikely to be responsible for the absence of H_2_S-induced hypothermia in our experiments. A ‘masked’ thermoregulatory vasodilative response would be accompanied by deactivation of brown adipose tissue (BAT) and shivering thermogenesis (i.e., major source of heat in mice at a *T*_a_ of 21 °C)^1,39^. Subsequently, the cessation of thermogenesis would be reflected in the *T*_b_/superficial temperature of H_2_S-exposed animals, which was not observed (Fig. 3).

In conclusion, exogenous H_2_S is not a hypometabolism-inducing agent. The hypometabolism induced in mice that were subjected to exogenous H_2_S was caused by hypoxia. At subatmospheric *F*_i_O_2_ levels, exogenous H_2_S exacerbates the hypoxic conditions to such a degree that anapyrexia and hypothermia are triggered. Accordingly, exogenous H_2_S is a hypometabolic adjuvant rather than a hypometabolism-inducing agent.

## Materials and Methods

### Animals

Forty-eight female C57Bl/6 mice (Charles River, L’Arbresle, France; 10–12 weeks of age) were acclimated for 2 weeks under standardized laboratory conditions with a 12 h light/dark cycle, a constant ambient temperature (*T*_a_) of approximately 21 °C, and ad libitum access to standard chow and drinking water. The experimental protocol was evaluated and approved by the animal ethics and welfare committee of the Academic Medical Center, University of Amsterdam under protocol number BEX 102753. Animals were treated in compliance with institutional guidelines and the *National Institute of Health Guidelines for the Care and Use of Laboratory Animals* (NIH publication No. 86–23, revised 2011).

### Experimental setup and gas mixtures

The hydrogen sulfide (H_2_S) and 17% oxygen (O_2_) gas mixtures were obtained from Westfalen (Münster, Germany) and consisted of (1) 80 ppm H_2_S, 21% O_2_, and 79% nitrogen (N_2_); (2) 80 ppm H_2_S, 17% O_2_, and 83% N_2_; or (3) 17% O_2_, and 83% N_2_. The 5% O_2_ gas mixture was obtained from Linde Gas (The Linde Group, Munich, Germany) and consisted of 5% O_2_ and 95% N_2_. Normo-atmospheric air (21% O_2_ and 79% N_2_) was used as control.

**Figure 5:**
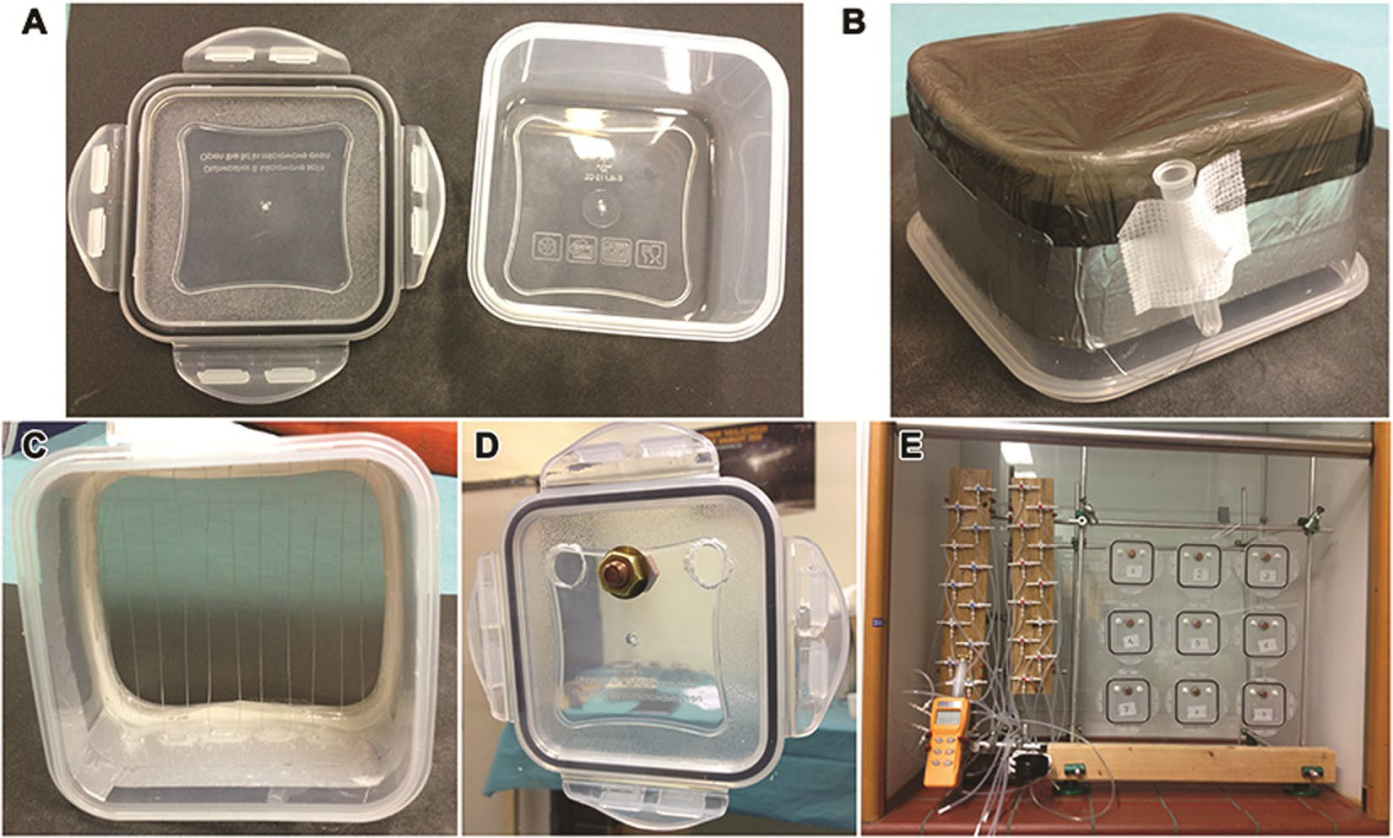
Components of the experimental setup. (**A**) The bare polypropylene chamber, consisting of the main chamber and the lid (back part of the chamber). (**B**) The bottom of the main chamber was removed and replaced with a polyethylene sheet. (**C**) Metal wires were inserted in front of the polyethylene sheet at a distance at which the mice could not pass through. (**D**) A bolt was inserted into the back panel to measure the actual temperature in the chamber. The bolt was connected to a thermistor. (**E**) Configuration of the tubing that was connected to the different gas-containing cylinders and used to modulate the chamber atmosphere during the experiments.

An experimental setup was custom-built to allow controlled gas exposure while unobtrusively assessing body temperature (*T*_b_) with a thermographic camera (ThermaCAM SC2000, FLIR Systems, Wilsonville, OR) in non-anesthetized mice. The setup consisted of gas-tight polypropylene chambers (Fig. 5A, length × depth × height of 109 mm × 109 mm × 61 mm) that were sealed at the imaging end with a thin, infrared light-permeable polyethylene sheet to permit thermal imaging from outside (Fig. 5B). Metal wires were secured longitudinally so that the animals could not reach the polyethylene sheet (Fig. 5C). Gas inflow and outflow tubes were connected to each box at the posterior end for modulation of experimental conditions (Fig. 5E). The gas permeability of the chambers was tested by air pressure decline experiments. Also, a thermistor (Fluke 51 II, Fluke Corporation, Everett, WA) was secured in the posterior wall (Fig. 5D) to facilitate the measurement of the temperature in the chamber. The thermistor was used as a calibrator for the thermographic camera images, as the thermographic images display the temperature of the copper bolt retaining the thermistor.

To ascertain sufficient inflow of gas in all experiments and prevent CO_2_ accumulation, the flow rates were controlled on the basis of CO_2_ outflow concentrations (<600 ppm, CO_2_ Meter, Ormond Beach, FL). The system was also connected to an O_2_ and H_2_S meter (model OdaLog 7000, App-Tek International, Brendale, Australia), which was calibrated by a certified company prior to the experiments (Carltech, Maarheeze, the Netherlands). The O_2_ and H_2_S meter was post hoc tested for measurement accuracy. The experiments were performed at a mean ± SD *T*_a_ of 21.2 ± 0.6 °C.

### Experimental procedure

To test the hypothesis that H_2_S-induced hypothermia emanates from hypoxia and not H_2_S, all 48 animals were randomly divided among 5 experimental groups. Group A was exposed to 80 ppm H_2_S in 21% O_2_ and 79% N_2_ (H_2_S in 21% O_2_ group, N = 12), group B was exposed to 80 ppm H_2_S in 17% O_2_ and 83% N_2_ (H_2_S in 17% O_2_ group, N = 6), group C was exposed to 5% O_2_ and 95% N_2_ (5% O_2_ group, N = 12), group D was exposed to 17% O_2_ and 83% N_2_ (17% O_2_ group, N = 6), and group E was exposed to 21% O_2_ and 79% N_2_ (normoxia group, N = 12).

Mice were placed in the chambers individually. After 1 h of exposure to normoxia (21% O_2_ and 79% N_2_), the mice were exposed to one of the gas mixtures (A – E) for 6 h, after which 6 of the animals per group were allowed to recover at normoxic conditions for 3 h before being terminated. The other 6 animals of group A, C and E were terminated immediately after the 6 h of exposure for another study.

No anesthetics were used before or during the experimental procedure.

### Thermal imaging and data processing

Animals were filmed every hour for 10 min with a thermographic camera (ThermaCAM SC2000, FLIR Systems, Wilsonville, OR)(Fig. 2). Thermographic camera images (3 images per second) were processed and analyzed in ThermaCAM Researcher 2001 (FLIR Systems). The mean maximum superficial temperature was calculated per time point per group.

The tail temperatures of animals in group A (N = 3), C (N = 3), and E (N = 1) were obtained from the thermographic camera images at 0 h, just before the start of exposure, and approximately 4 min after the start of exposure. We noticed the intergroup differences in tail temperature during the experiments, as a result of which the tail temperature was measured in only 7 animals. The mean difference in tail temperature between both time points was calculated and compared for group A and C.

Locomotor activity was assessed per time point using the same thermal images as were used for the calculation of superficial temperature. An analytics program was written in LabVIEW (LabVIEW, National Instruments, Austin, TX). The thermographic camera images were converted to grayscale images and loaded into LabVIEW. Locomotor activity was calculated per animal per time point (−1 up to 9 h) on the basis of fluctuations in pixel intensity. A pixel was considered to reflect ‘motion’ when the grayscale intensity difference between direct temporally consecutive pixels exceeded 7 on a scale of 0 to 255. The intensity difference of at least 7 was based on the disappearance of background scatter present as intensity differences between 1 and 6. Values were expressed as the mean ± SEM amount of pixels with ‘motion’ per group per time point.

### Statistical analysis

Statistical analyses were performed using MatLab 2013a (MathWorks, Natick, MA). Homogeneity of variance in each group was tested using the Bartlett’s test. Based on equality of variances, either a one-way ANOVA or a Kruskal-Wallis test was performed, followed by a Tukey’s range test or Dunn’s test, respectively, to compare ordinal variables related to maximum superficial temperature and locomotor activity between groups. Tail temperature values were compared using an unpaired student’s *t*-test. *P*-values less than 0.05 were considered significant. All values were presented as mean ± SEM, unless otherwise mentioned.

## Electronic supplementary material

Supplemental Video S1

Supplemental Video S1 Legend
