# Supplementary material for: Exogenous hydrogen sulfide gas does not induce hypothermia in normoxic mice
Source: Sci Rep. 2018 Mar 1;8:3855. doi: 10.1038/s41598-018-21729-8 (PMC5832815; doi:10.1038/s41598-018-21729-8)
Supplement: Supplementary file 2 — Supplemental Video S1 Legend [file 41598_2018_21729_MOESM2_ESM.doc]

**SUPPLEMENTAL INFORMATION**

**Exogenous hydrogen sulfide gas does not induce hypothermia in normoxic mice**

Sebastiaan D. Hemelrijk*^1$^*, Marcel C. Dirkes*^1,2$^*, Marit H.N. van Velzen*^3^*, Rick Bezemer*^2,4^*, Thomas M. van Gulik*^1^*, Michal Heger*^1^*^*^

*^1^ Department of Experimental Surgery, Academic Medical Center, University of Amsterdam, Amsterdam, the Netherlands*

*^2^ Philips Research, Eindhoven, the Netherlands*

*^3^ Department of Anesthesiology, Laboratory of Experimental Anesthesiology, Erasmus University Medical Center Rotterdam, the Netherlands*

*^4^ Department of Translational Physiology, Academic Medical Center, University of Amsterdam, Amsterdam, the Netherlands*

**Supplemental Video S1 legend**

Composite video of representative mice exposed to a normoxic H_2_S atmosphere (80 ppm H_2_S and FiO2 21%, left panel), a hypoxic atmosphere (FiO2 5%, central panel), and a normoxic atmosphere (FiO2 21%, right panel) at different time points in the experiment (see Figure 2 in the main text for experimental detail). The video clearly shows that the H_2_S gas-subjected mouse does not enter a state of hypothermia, as exhibited by the hypoxia-exposed mouse. Consequently, induction of a hypometabolic state in mice requires a hypoxic atmosphere, and not exposure to H_2_S alone (under normoxic conditions).
